# Intranasal Administration of Catechol-Based Pt(IV) Coordination Polymer Nanoparticles for Glioblastoma Therapy

**DOI:** 10.3390/nano12071221

**Published:** 2022-04-05

**Authors:** Xiaoman Mao, Pilar Calero-Pérez, David Montpeyó, Jordi Bruna, Victor J. Yuste, Ana Paula Candiota, Julia Lorenzo, Fernando Novio, Daniel Ruiz-Molina

**Affiliations:** 1 Catalan Institute of Nanoscience and Nanotechnology (ICN2), CSIC and BIST, Campus UAB, Cerdanyola del Vallès, 08193 Barcelona, Spain; mmchong7@zju.edu.cn (X.M.); dani.ruiz@icn2.cat (D.R.-M.); 2 Centro de Investigación Biomédica en Red: Bioingeniería, Biomateriales y Nanomedicina, Cerdanyola del Vallès, 08193 Barcelona, Spain; pilar.calero@uab.cat; 3 Departament de Bioquímica i Biologia Molecular, Universitat Autònoma de Barcelona (UAB), Cerdanyola del Vallès, 08193 Barcelona, Spain; victor.yuste@uab.cat; 4 Institut de Biotecnologia i de Biomedicina, Universitat Autònoma de Barcelona (UAB), Cerdanyola del Vallès, 08193 Barcelona, Spain; david.montpeyo@uab.cat; 5 Neuro-Oncology Unit, Bellvitge University Hospital-ICO (IDIBELL), Avinguda de la Gran Via de l’Hospitalet, 199-203, L’Hospitalet de Llobregat, 08908 Barcelona, Spain; jbruna@bellvitgehospital.cat; 6 Institut de Neurociències, Facultat de Medicina, Universitat Autònoma de Barcelona (UAB), Campus UAB, Cerdanyola del Vallès, 08193 Barcelona, Spain; 7 Departament de Química, Universitat Autònoma de Barcelona (UAB), Campus UAB, Cerdanyola del Vallès, 08193 Barcelona, Spain

**Keywords:** nanoscale coordination polymers, platinum, catechol, intranasal administration, glioblastoma, preclinical studies, GL261 GB, magnetic resonance imaging

## Abstract

Cisplatin has been described as a potent anticancer agent for decades. However, in the case of glioblastomas, it is only considered a rescue treatment applied after the failure of second-line treatments. Herein, based on the versatility offered by coordination chemistry, we engineered nanoparticles by reaction of a platinum (IV) prodrug and iron metal ions showing in vitro dual pH- and redox-sensitivity, controlled release and comparable cytotoxicity to cisplatin against HeLa and GL261 cells. In vivo intranasal administration in orthotopic preclinical GL261 glioblastoma tumor-bearing mice demonstrated increased accumulation of platinum in tumors, leading in some cases to complete cure and prolonged survival of the tested cohort. This was corroborated by a magnetic resonance imaging follow-up, thus opening new opportunities for intranasal glioblastoma therapies while minimizing side effects. The findings derived from this research showed the potentiality of this approach as a novel therapy for glioblastoma treatment.

## 1. Introduction

Glioblastoma (GB) is one of the most common and lethal malignant brain tumors. The median survival after diagnosis is 12–18 months, and the five-year survival rate is less than 5% [1]. Standard treatment includes surgical resection followed by radiotherapy and concomitant and adjuvant chemotherapy with temozolomide (TMZ) [2,3]. Unfortunately, only a small fraction of the administered TMZ reaches the brain tissue, mostly due to the low permeability of the blood-brain barrier (BBB) [4], even in cases with compromised BBB such as GB. Recently, several strategies have been investigated to overcome this challenge by increasing BBB permeability [5,6,7], allowing drug delivery within biodegradable polymer implants in the tumor bed [8,9,10], or mediated by simple diffusion using a reservoir-catheter system or positive pressure bulk flow via convection-enhanced delivery (CED) [11,12]. However, most of these approaches require a neurosurgical intervention at the relapsing tumor scenario or device placement with the consequent problems both in neuroimaging response and surgical complications risk. Therefore, there is an urgent need to develop new opportunities for pharmaceutical applications beyond oral, intravenous, or alternative administration routes.

**Figure 1:**
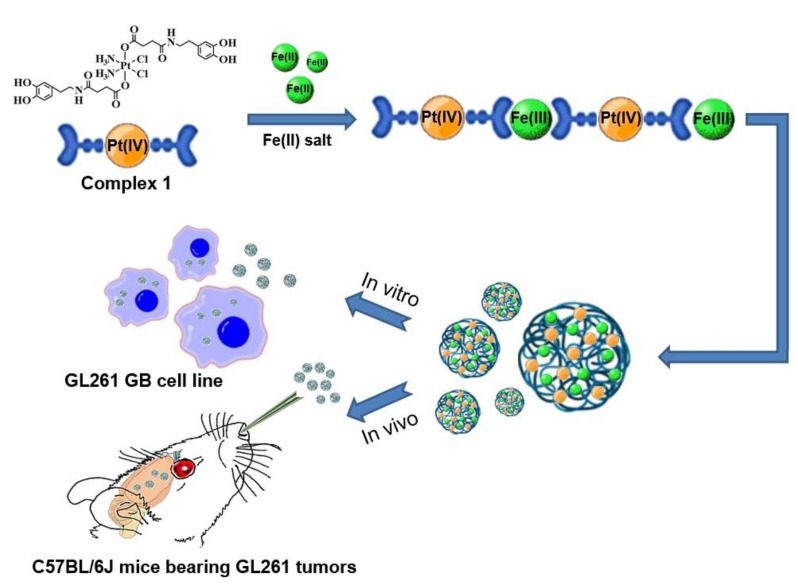
Scheme of the Pt-Fe NCPs synthesis upon polymerization of complex **1** with iron ions as metal nodes in open to air conditions, and analysis of their therapeutic effect in vitro (GL261 cell line) and in vivo murine models (C57BL/6J mice bearing orthotopic GL261 GB tumors).

As alternative to TMZ second-line treatments based on nitrosourea-based drugs [13,14], anti-angiogenic agents [15], topoisomerase inhibitors [16] or even platinum complexes [17,18] have been used. Unfortunately, some of these developments do not improve half-life and toxicity or require the use of therapeutic concentrations well over the tolerated dose-limiting toxicity. Interestingly, most of these restrictions may be overcome, at least partially, with the use of nanoparticles [19,20]. Additionally, the nose-to-brain or intranasal (IN) route has emerged as a non-invasive and easily accessible approach that has been explored in the clinical phase within the last decade (e.g., NCT04091503, NCT02704858) [21,22,23]. In addition to bypassing the BBB, IN-administered drugs can be rapidly absorbed through rich vasculature in the submucosa, exhibiting a fast onset of action while minimizing side effects [24,25]. This can give a second chance to other drugs beyond TMZ, such as platinum complexes mostly used nowadays in GB only when all standard therapeutic protocols fail. For instance, we have recently described a bioinspired Pt(IV) prodrug functionalized with catechol moieties which enhances the in vivo intranasal mucopenetration, inhibiting the growth of GL261tumors [26]. However, and in spite of this and other efforts, to the best of our knowledge, no intranasal drugs for GB, and even fewer platinum-based therapies, are commercialized or going through advanced clinical phase trials. Herein we hypothesize that nanostructured coordination polymers (NCPs) containing Pt(IV) prodrugs as constitutive building blocks represent an excellent scenario to achieve this goal (see Figure 1).

The reason is threefold. First, in a very recent publication, our research group has demonstrated how these NCPs can reach the brain and effectively release drugs upon intranasal administration [27]. Second, Pt(IV)-prodrugs have already been used as building blocks to obtain NCPs with high payloads and effective controlled release [28]. Finally, last but not least, Pt(IV) prodrugs are less toxic and only become active after intracellular reduction to the Pt(II) form, minimizing side effects and improving the pharmacological properties [29,30,31]. As a proof-of-concept, we have obtained Pt(IV)-based nanoparticles (from now on Pt-Fe NCPs) by reaction of complex **1** bearing bis-catechol groups and iron ions acting as metal nodes of the resulting polymer. These nanoparticles have been analyzed to determine the Pt(IV) encapsulation yields and subsequent controlled release, together with the study of their in vitro therapeutic efficacy in comparison with cisplatin when tested in murine GB cells. On top of that, in vivo tolerability and therapeutic activity have been determined for the evaluation of Pt-Fe NCPs as efficient IN nanoformulations for treating GB.

## 2. Materials and Methods

### 2.1. Materials

Solvents and starting materials were purchased from Sigma–Aldrich (Madrid, Spain) and used as received, without further purification, unless otherwise stated. Complex **1** (Pt(IV) prodrug) was synthesized according to the previously reported methodology [14].

### 2.2. Synthesis of Pt-Fe NCPs

In order to prepare a material suitable for biological experiments, nanoparticle synthesis was carried out inside a biosafety cabinet (Telstar BioVanguard B green), and all materials and solvents were sterilized by autoclaving prior to their use. Complex **1** (50 mg, 0.06 mmol) was dissolved in 15 mL of 96% ethanol (EtOH), and iron acetate (Fe(OAc)_2_) (10.8 mg, 0.06 mmol) in 1 mL of H_2_O was added dropwise. A black solid in suspension was formed immediately with the addition of iron ions. After 30 min stirring at 700 rpm, the precipitate was collected by centrifugation (12,000 rpm) and then washed with ethanol and water several times. The solid was then lyophilized to get a black powder which was stored in the freezer for later use. Elemental analysis: found (%) C 28.63, H 2.54, N 5.79. Metal contents by ICP-MS: 18.87% Pt, 15.13% Fe.

### 2.3. Characterization Methods

Spectra at 400 MHz ^1^H NMR, ^1^H–^1^H COSY, and 100 MHz ^13^C NMR were recorded on a Bruker DPX 400 MHz spectrometer (Bruker France S.A.S., Wissembourg, France). Chemical shifts (δ) are given in ppm, using the residual non-deuterated solvent as the internal reference. High-resolution mass spectra were obtained by direct injection of the sample with electrospray techniques in a Bruker microTOF-Q instrument. FT-IR spectra were recorded using a Tensor 27 spectrophotometer (Bruker Optik GmbH, Ettlingen, Germany) with KBr pellets. Powder X-ray diffraction (PXRD) patterns were recorded at room temperature on a PANalytical X’Pert PRO MRD diffractometer (Malvern PANalytical, Kassel, Germany) equipped with a CuKα radiation source (λ = 1.54184 Å) and operating in reflection mode, where the solid samples were placed on an amorphous silicon oxide plate and measured directly. UV-vis analysis was performed using a Cary 4000 UV-vis spectrometer (Agilent Technologies, Santa Clara, CA, USA) within a wavelength range of 175–900 nm and a 1 cm path length quartz cuvette (QS 10 mm). Size distribution and surface charge of the nanoparticles (0.5 mg/mL) were measured by Dynamic Light Scattering (DLS), using a ZetasizerNano 3600 instrument (Malvern Instrument, Malvern, UK), with a size range limit from 0.6 nm to 6 nm. The samples were comprised of aqueous dispersions of nanoparticles in distilled water or in Phosphate Buffered Saline (PBS) buffer. All samples were diluted to obtain adequate nanoparticle concentrations. Scanning Electron Microscopy (SEM) images were obtained using a scanning electron microscope (FEI Quanta 650 FEG, Hillsboro, OR, USA) at acceleration voltages of 5–20 kV. The SEM samples were prepared by drop-casting of corresponding NPs dispersions on an aluminum tape followed by evaporation of the solvent under room conditions. Before analysis, the samples were metalized with a thin layer of platinum (thickness: 5 nm) using a sputter coater Emitech K550 (Emitech, Montigny-le-Bretonneux, France). Scanning transmission electron microscopy (STEM) images were obtained using a scanning-transmission electron microscope (Magellan 400 L, FEI), while TEM images were recorded using a transmission electron microscope (Tecnai G2 F20, FEI) at a voltage of 200 kV. Samples for STEM and TEM were prepared by drop-casting corresponding dispersions on TEM grids (ultrathin carbon type-A, 400 mesh Cu grid, Ted Pella Inc., Redding, CA, USA) and drying overnight under room conditions prior to examination. ^57^Fe Mössbauer spectroscopy measurements were recorded in transmission geometry with a conventional Mössbauer spectrometer equipped with a ^57^Co(Rh) radioactive source operating at room temperature 300 K and relatively low temperature 80 K. The samples were sealed in aluminum foil and mounted on a nitrogen bath cryostat (Oxford Instruments, Abingdon, UK). The spectra were fitted to the sum of Lorentzians by a least-squares refinement using Recoil 1.05 Mössbauer Analysis Software. All isomer shifts refer to α-Fe at room temperature. Inductively coupled plasma-mass spectrometry (ICP-MS) was performed using an ICP-MS NexION 300× (Perkin Elmer, Waltham, MA, USA). Isotopes of In and Cu were selected as tracers. All samples were measured in quadruplicate in an argon atmosphere and subsequently analyzed with ICP NexION version 8.2 software (PerkinElmer). The metal concentration for each sample was calculated using a calibration curve using the National Institute of Standards and Technology (NIST)-traceable 1000 mg/L elemental standards (PerkinElmer Inc., Rodgau, Germany) in the range of 0.01−250 ppb of metal. The isotopes ^54^Fe and ^57^Fe were selected as Fe tracers, while ^194^Pt, ^195^Pt, and ^196^Pt as Pt tracers.

### 2.4. In Vitro Magnetic Resonance Imaging Analyses (MRI-Phantoms)

The longitudinal r1 and transverse r2 relaxation rates for different concentrations of Pt-Fe NCPs were measured in solution under an external magnetic field at 7 T (300 MHz) BioSpec 70/30 USR spectrometer (Bruker BioSpin GmbH, Ettlingen, Germany) with corresponding acquisition sequences. Specifically, the Pt-Fe NCPs were dispersed in 1.5 mL of 1% agarose/PBS solutions at 65 °C in an ultrasound bath in order to ensure a good colloidal suspension, and solidified at room temperature, resulting in a series of gel with different metal concentrations (3.94, 1.97, 1, 0.1, and 0 mM of Fe). The obtained relaxation rate values were plotted versus the concentrations of iron.

### 2.5. Drug Release Assay

Drug release from Pt-Fe NCPs was determined using a dialysis method. Pt-Fe NCPs were redispersed in 1 mL of PBS buffer in a dialysis bag (Molecular weight cutoff (MWCO) = 6000–8000) at 1 mg/mL. The dialysis tubes were immersed into sealed beakers with 40 mL of PBS buffer adjusted at pH values of 7.4 and 5.5. The beakers were incubated at 37 °C with gentle stirring during the study. Aliquots of 500 μL were taken from the dialysate at predetermined time points (0.5, 1, 2, 4, 6, 8, 24, and 48 h), followed by supplementing it with the same volume fresh buffer immediately. The amount of released Pt was determined by ICP-MS. To test the influence of glutathione (GSH), PBS buffers containing 2 mM or 10 mM GSH were used for dialysis, while the remaining conditions were kept unchanged.

### 2.6. Cell Lines Culture

The human cervical cancer HeLa cell line was purchased from the American Type Culture Collection (ATCC, Manassas, VA, USA), while the GL261 glioblastoma cell line was purchased from the Tumor Bank Repository at the National Cancer Institute (NCI, Frederick, MD, USA). HeLa cells were routinely cultured in Minimum Essential Medium (MEM) and GL261 cells in Roswell Park Memorial Institute (RPMI 1640) medium. Both culture media were supplemented with 10% heat-inactivated fetal bovine serum (FBS, Gibco^®^, Invitrogen, Inchinnan, UK) and 1% penicillin-streptomycin. Cell lines were cultured at 37 °C in a humidified atmosphere of 95% O_2_ and 5% CO_2_. RPMI 1640. The antibiotics, trypsin and Trypan Blue, were purchased from Sigma-Aldrich (Madrid, Spain). Other reactants were purchased from Fisher Scientific (Thermo Scientific, Waltham, MA, USA).

### 2.7. In Vitro Cytotoxicity Assays

The cells (HeLa, GL261) in exponential growth were seeded in a 96-well plate (Corning, Tewksbury, MA, USA) at a proper density. HeLa cell line was seeded at a density of 2000 cells/well, and GL261 at 4000 cells/well. After 24 h of incubation, fresh media containing compounds (Pt-Fe NCPs, complex **1**, and cisplatin) at different concentrations (0, 0.1, 1, 5, 10, 25, 50, 100, 200 μM referred to Pt concentration) were added, and plates were incubated for 24 or 72 h. Afterwards, 10 μL of PrestoBlue^®^ (0.15 mg/mL, Thermo Scientific, Waltham, MA, USA) was added to each well, and plates were incubated for another 4 h before measuring the fluorescence at 572 nm with excitation at 531 nm by the microplate reader Victor 3 (Perkin Elmer). All experiments were carried out in triplicate, and data were treated and calculated with GraphPad Prism software (version 7.0, San Diego, CA, USA) to obtain IC_50_.

### 2.8. Cellular Internalization Studies

HeLa and GL261 cells were seeded in 6-well plates (Corning) at a density of 300,000 cells per well with 1.5 mL of media. After 24 h of incubation, media were replaced by 1 mL of fresh media with or without cisplatin, complex **1**, and Pt-Fe NCPs at 10 μM referred to Pt concentration. Treated cells were allowed to internalize the compounds at increasing time points of 2, 4, 8, and 24 h. The media were removed immediately, and cells were rinsed twice with cold PBS in order to remove non-internalized compounds. Then, 0.5 mL of trypsin was added to each well for 3 min, and a 2-fold volume of fresh media was added to neutralize the trypsin. After that, an aliquot was taken from each well for cell counting, and the remaining cell suspensions were collected into 1.5 mL Eppendorf tubes (Corning) and centrifuged at 12,000 rpm for 4 min. The supernatants were discarded while cell pellets were stored at −80 °C for further quantification by ICP-MS.

### 2.9. DNA-Bound Pt

In order to clarify the mechanism of action of our Pt NPs, DNAs were extracted and measured by ICP-MS in order to quantify the DNA-bound Pt after 24 h uptake for HeLa and GL261 cells. Cells in exponential growth were seeded onto cell culture dishes in optimal conditions for each cell line to reach 50–60% of confluence in 24 h before treatment. Then, the drugs (cisplatin, complex **1**, and Pt-Fe NCPs) were added at 10 μM referred to Pt concentration, and cells were allowed to incubate for another 24 h. Afterwards, media with drugs were removed, and cells were rinsed, trypsinized, and collected by centrifugation, being further rinsed twice with cold PBS to remove the remaining drugs in solution. The resulting cell pellets were resuspended in lysis buffer (pH 8.0, 150 mM Tris-HCl, 100 mM NaCl and 0.5% (*w*/*v*) SDS). Pellets in buffer were incubated on ice for 15 min and centrifuged at 15,000 rpm for 15 min. Later on, 0.1 volume of RNase A was added to each supernatant at 200 μg/mL and incubated for 1 h at 37 °C. Afterwards, Proteinase K was added at 100 μg/mL and incubated for 3 h at 56 °C. A volume of Phenol/Chloroform/Isoamyl alcohol (25:24:1 in volume, Thermo Scientific, Waltham, MA, USA) was added and mixed gently. After centrifugation for 3 min at 15,000 rpm, aqueous phases containing DNA were transferred into sterile tubes. DNA was precipitated with 0.1 volume of 3 M sodium acetate and 1 volume of absolute ethanol at −20 °C overnight. Then, DNA samples were centrifuged for 15 min at 15,000 rpm, and finally, DNA samples were dried and resuspended in 100 μL of elution buffer (pH 8.0, 10 mM Tris-HCl, 1 mM EDTA). The concentration of isolated DNA was quantified by measuring the absorbance at 260 nm using NanoDropTM 1000 spectrophotometer (Thermo Fisher Scientific, Waltham, MA, USA). Samples were kept frozen for further ICP-MS analyses.

### 2.10. Sample Digestion Treatments for ICP-MS Measurement

All glassware was immersed in 20% HNO_3_ for 48 h, and plastic ware in 5% HNO_3_ for 4 h prior to their use for ICP-MS. All samples were digested using the wet digestion method. Depending on the materials to digest, different procedures were adopted.

Chemical samples: samples, 5.0 mg of NPs, for instance, were weighed accurately with an analytical balance and transferred into a vial, then concentrated ultrapure HNO_3_ (69%, Ultratrace^®^, ppb-trace analysis grade, Scharlab, Barcelona, Spain) was added. The samples were placed in the fume hood for 48 h to be fully digested, then were diluted with 0.5% (*v*/*v*) ultrapure HNO_3_ for further measurements.

Cell pellets: cell pellets were immersed in 100 µL of concentrated ultrapure HNO_3_ (69%, Ultratrace^®^, ppb-trace analysis grade, Scharlab) and left to digest overnight. Samples were then heated to 90 °C until suspensions turned clear. The samples were diluted with 0.5% (*v*/*v*) ultrapure HNO_3_ to appropriate volumes for later measurements.

Tissues: tissues were added with Tissue Protein Extraction Reagent buffer (T-PER^TM^, Thermo Fisher Scientific, Waltham, MA, USA) in a ratio of 10 mL:1 g, then cut into small pieces and homogenized with ultrasonication microtip (Branson Digital Sonifier 450, Emerson, St. Louis, MO, USA) in cycles of 10 s on and 15 s off with an amplitude of 30%. Afterwards, the tissue suspensions were added with aqua regia (all ppb-trace analysis grade) and heated up to 300 °C, while 30% H_2_O_2_ was added in the later digestion process until the suspensions turned clear. The clear solutions were transferred and added with 0.5% (*v*/*v*) ultrapure HNO_3_ to appropriate volumes for the determination of metal contents using ICP-MS.

### 2.11. Estimation of ROS Formation

GL261 cells were seeded into black 96-well plates (Corning) at a density of 20,000 cells per well. After 24 h incubation, the medium was discarded, cells were washed out, and added to pre-warmed PBS with the fluorescent probe 2′,7′-dichlorofluorescin diacetate (DCFCDA, final working concentration 10 μM), then incubated for 30 min. After that, the buffer was replaced with only medium or media with compounds (H_2_O_2_, Fe(OAc)_2_, Pt-Fe NCPs, complex **1**, and cisplatin at the concentration of IC_50_) for 24 h. Then, 0.1 mM of H_2_O_2_ was added as a positive control, while 0.1 mM of Fe(OAc)_2_ was added for comparison purposes. After 24 h, the fluorescence of each well was examined at 530 nm after excitation at 485 nm by a microplate reader Victor 3 (Perkin Elmer). This experiment was repeated in triplicate independently. The results were normalized based on the negative control loaded with dye but without drug treatment.

### 2.12. Animal Studies

Animal experiments and handling have been performed in collaboration with properly accredited personnel. Healthy female C57BL/6J mice (8–12 weeks, body weight 20–24 g) were used for in vivo studies in this work. Mice were obtained from Charles River Laboratories (Charles River Laboratories Internacional, L’Abresle, France) and housed in the UAB animal facility (Servei d’Estabulari, https://estabulari.uab.cat/; accessed on 24 March 2022) of the Universitat Autònoma de Barcelona. All animal study protocols were approved by the local ethics committee (Comissió d’Ètica en l’Experimentació Animal i Humana, https://www.uab.cat/etica-recerca/; accessed on 24 March 2022) according to regional and state legislation (protocol CEEAH-4859). The animals were housed in cages with free access to standard food and water, under uniform housing and environmentally controlled conditions.

### 2.13. GL261 GB Preclinical Model Generation and Animal Treatment

#### 2.13.1. Tumor Generation

The orthotopic GB tumor-bearing mice were generated through stereotactic injection of GL261 GB cells into C57BL/6J mice striatum. Specifically, analgesia (Metacam, Boehringer Ingelheim, Ingelheim am Rhein, Germany) at 1 mg/kg was injected subcutaneously into each animal 15 min before anesthesia and also 24 and 48 h after implantation. Mice were anesthetized with a mixture of ketamine (Parke-Davis SL, Madrid, Spain) at 80 mg/kg and xylazine (Carlier, Barcelona, Spain) at 10 mg/kg via intraperitoneal administration. Once anesthetized, the mice were immobilized on the stereotaxic holder (Kopf Instruments, Tujunga, CA, USA) in a prone position. Next, the head area was shaved, and the incision site was sterilized with iodophor disinfectant solution, a 1 cm incision was made exposing the skull, and a 1 mm hole was drilled 0.1 mm posterior to the bregma and 2.32 mm to the right of the midline using a microdrill (Fine Science Tools, Heidelberg, Germany). A 26G Hamilton syringe (Reno, NV, USA), positioned on a digital push-pull microinjector (Harvard Apparatus, Holliston, MA, USA), was then used for the injection of 4 µL of RPMI 1640 cell culture medium containing 100,000 GL261 cells at a depth of 3.35 mm from the surface of the skull at a rate of 2 µL/min. Once the injection was completed, the Hamilton syringe was left untouched for 2 min more before its removal to prevent the cellular liquid leakage out of the skull. Finally, the Hamilton syringe was gently and slowly withdrawn, and the scission site was closed with suture silk 6.0 (Braun, Barcelona, Spain). When the implantation was finished, mice were left in a warm environment to recover from anesthesia. The C57BL/6J immunocompetent mice were exposed to an enriched environment (EE) for 3 weeks before tumor implantation since it was reported that it could significantly reduce glioma growth and improve mice survival by increasing immunological parameters in the brain of mice [32]. Moreover, since this is the current protocol used at Grup d’Aplicacions Biomèdiques de la Ressonància Magnètica Nuclear (GABRMN), the maintenance of basic parameters may ensure proper comparisons.

#### 2.13.2. Tumor-Bearing Mice Treatment

For intranasal administration, animals were anesthetized with isoflurane for 1 min and kept in a supine position. The animals were administered with 2 µL of formulation for each nostril, with an interval of about 2 min between each administration, following an immune-friendly schedule, every 6 days, as described in Section 3.8. Treated animals were followed-up with MRI (Section 2.15) for evaluating tumor volume evolution. Mice reaching endpoint criteria were euthanized for humanitarian reasons.

### 2.14. Tissue Preservation Procedures

When animals died or were euthanized by cervical dislocation to prevent suffering, the brains and main organs (heart, lungs, spleen, liver, and kidneys) were excised and either frozen in liquid nitrogen or fixed in formalin, depending on the purpose. In case of freezing, tumors were dissected and isolated from normal brain parenchyma, frozen in a liquid nitrogen container for further analysis. In case of fixation, tissue (or the whole animal) was preserved in 4% formalin for further histopathological analysis or autopsy. Tissues were resected after visual inspection of the whole brain and tumor, avoiding cross-contamination with non-tumoral tissue as much as possible.

### 2.15. In Vivo MRI Studies

MRI acquisitions were performed in GL261 GB-bearing mice in order to measure tumor location and volume evolution. Studies were carried out at 7T BioSpec 70/30 USR spectrometer (Bruker BioSpin GmbH, Ettlingen, Germany) at Servei de Ressonància Magnètica from UAB (also part of Unit 25 of ICTS NANBIOSIS, NMR: Biomedical Applications I https://www.nanbiosis.es/portfolio/u25-nmr-biomedical-application-i/; accessed on 24 March 2022). Mice were positioned in a dedicated bed, which allowed the delivery of anesthesia (isoflurane, 1.5–2% in O_2_ at 1 L/min), with an integrated heating circuit for body temperature regulation. Respiratory frequency was monitored with a pressure probe and kept between 60–80 breaths/min. T2-weighted (T2w) MRI was acquired using a rapid acquisition with relaxation enhancement (RARE) sequence. The acquisition parameters were as following: repetition time (TR)/effective echo time (TEeff) = 4200/36 ms; echo train length (ETL) = 8; field of view (FOV) = 19.2 mm × 19.2 mm; matrix size (MTX) = 256 × 256 (75 µm/pixel × 75 µm/pixel); number of slices (NS) = 10; slice thickness (ST) = 0.5 mm; inter-ST = 0.1 mm; number of averages (NA) = 4; total acquisition time (TAT) = 6 min and 43 s. MRI data were acquired and processed on a Linux computer using ParaVision 5.1 software (Bruker BioSpin GmbH, Ettlingen, Germany).

To calculate the tumor volume from MRI acquisitions, ParaVision software was used to generate regions of interest (ROIs) to measure the tumor area in each slice, and tumor volumes of the studied mice were calculated with the following equation:

TV (mm^3^) = [(AS_1_ × ST) + [(AS_2_ + (…) + AS_10_) × (ST + IT)]] × 0.075^2^ (1)

where TV is the tumor volume; AS is the number of pixels contained in the ROI delimited by the tumor boundaries in each slice of the MRI sequence; ST is the slice thickness (0.5 mm), while IT is the inter-slice thickness (0.1 mm), and 0.075^2^ is the individual pixel surface area in mm^2^.

### 2.16. Tolerability Assays

Healthy female C57BL/6 mice (8–12 weeks, 20–24 g) were randomly divided into three groups, control, complex **1**, and Pt-Fe NCPs, *n* = 3 each. After one min of being anesthetized, mice in the supine position were intranasally administered with drugs using a micropipette (Gilson, Limburg, Germany). The drug dosage increased from 0.9 to 1.2 and 1.5 mg Pt/kg body weight every week, these being the scaling dosages based on the approach previously described [33]. The body weights of mice were recorded prior to the first administered dose and were monitored 3 times a week thereafter. The animals were inspected by veterinary staff from the UAB animal facility, in particular for inspecting abnormal behavior, food and water consumption, and possible suffering clinical signs [34]. The whole study lasted for 4 weeks. Mice were euthanized through cervical dislocation, and bodies were preserved either in liquid nitrogen or in formalin in case of further histological study.

### 2.17. In Vivo Biodistribution Study

GL261 GB-bearing mice were used for the biodistribution study. Pt-Fe NCPs were intranasally administered at a dose of 1.5 mg Pt/kg body weight. After 1 h, the mice were euthanized. Brain, tumor, heart, lungs, spleen, liver, and kidneys were excised and weighed immediately. These tissues were then homogenized in Tissue Protein Extraction Reagent (T-PER) buffer (10 mL/g tissue) using an ultrasound probe (Branson Digital Sonifier 450, Emerson) at an amplitude of 30%, with cycles of 10 s on and 15 s off. Afterwards, they were centrifuged at 10,000 rpm for 10 min. An aliquot was taken from each supernatant for total protein quantification. The remaining part was digested as described in the previous procedure and prepared for ICP-MS measurement in order to determine the metal concentration.

### 2.18. Assessment of Antitumor Efficacy in Vivo

The antitumor efficacy was evaluated in GL261 GB-bearing mice. All animals included in the study were confirmed to have localized intracerebral tumors with homogeneous volume using MRI T2w scanning as previously described. They were randomly divided into 2 groups: Control (untreated, *n* = 7), and Pt-Fe NCPs (*n* = 8). All formulations were given at a dose of 1.5 mg Pt/kg weight via intranasal administration, starting at day 10 (*n* = 3) or day 6 (*n* = 5) postimplantation, with the every-6-day schedule previously described in Section 2.13.2. Mice were declared cured when tumor mass disappeared after initial growth and it had turned into a stable tissue scar. In this case, treatment was halted, and animals were followed-up for suspicious signs/symptoms of tumor relapse, namely body weight loss or abnormal behavior.

### 2.19. Histological Examination

Necropsy of chosen Pt-administered mice from the tolerability study was performed by Unitat de Patologia Murina i Comparada (UPMiC, https://sct.uab.cat/upmic/es; accessed on 24 March 2022). The analysis was performed in *n* = 2 animals treated with complex **1** and *n* = 2 treated with Pt–Fe NCPs, and main organs (heart, lungs, liver, spleen, and kidneys) were examined.

### 2.20. Statistical Analyses

Unless otherwise stated, values are shown as average plus/minus standard error. A two-tailed Student’s *t*-test for independent measurements was used for comparisons. Comparisons of survival rates were performed with the log-rank test. The significance level for all tests was *p* < 0.05, while values comprised between 0.05 and 0.1 were considered a “trend to significance”.

## 3. Results

### 3.1. Synthesis and Characterization of Pt-Fe NCPs

**Figure 2:**
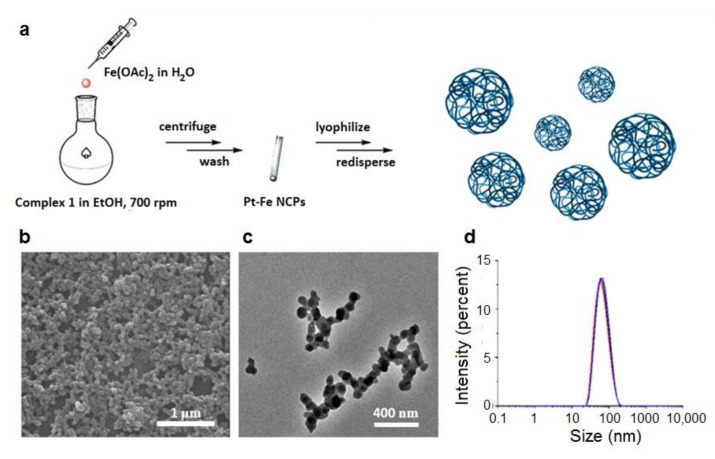
Pt-Fe NCPs: (**a**) Schematic illustration of the synthesis; (**b**) Representative SEM and (**c**) TEM images; (**d**) Representative size distribution obtained by DLS in PBS.

An Fe(OAc)_2_ salt was added over an ethanolic solution of complex **1**, obtained as previously described [14], using a 1:1 molar ratio and maintained under vigorous stirring, and open to air, until a dark-blue precipitate was observed. After centrifugation, the precipitate was collected and washed several times with ethanol and water to be finally lyophilized (49.3% yield). The dried nanoparticles could be redispersed in water and stored in the freezer. A schematic representation of the synthetic process is shown in Figure 2a, while synthetic details are given in the Experimental section. Scanning electron microscopy (SEM) and transmission electron microscopy (TEM) images of the resulting powder revealed the formation of monodisperse spherical nanoparticles with an average diameter of 70 ± 21 nm (Figure 2b,c), whereas dynamic light scattering (DLS) studies showed an average hydrodynamic radius of 66 ± 2.3 nm (PDI = 0.25) in water. The X-ray powder diffraction (XRD) pattern was characteristic of amorphous material (see Figure S1a). The energy-dispersive X-ray analysis (EDX) profile obtained from TEM qualitatively confirmed the presence of Pt and Fe in the polymeric material, along with C, O, and Cl (see Figure S1b) homogeneously distributed along the whole nanoparticle. Mössbauer spectroscopy studies confirmed that the iron was in the high-spin Fe(III) form (see Figure S2), suggesting that the initial Fe(II) used was oxidized along with the nanoparticle synthesis, most likely due to a redox reaction between the metal ion and the electroactive catechol ligands in the presence of oxygen, as previously reported [35]. A comparison of the FT-IR spectrum from Pt-Fe NCPs with the one from complex **1** showed noticeable changes, mainly in the catechol-based region (Figure S3), attributed to the coordination with the iron ions. More precisely, the characteristic band of the catechol peak at 1198 cm^−1^ decreased in intensity and shifted to 1220 cm^−1^, while peaks at 1527, 1328, and 1282 cm^−1^ disappeared. Moreover, two new intense peaks at 1484 cm^−1^ and 1261 cm^−1^ appeared, mostly associated with the C−O stretching of the catecholate moiety bonded to metal centers [36].

The correlation of C, H, and N elemental analysis (EA) and ICP-MS for platinum and iron content (see Table S1) resulted in a tentative chemical formula of [(Complex **1**)_1_Fe_3_(OH^−^)_5_]_n_ for Pt-Fe NCPs. This clearly differs from what was theoretically expected according to the initial ratio of reactants used (1:1, Fe:**1**). On the other hand, it is a well-known divergence for this family of NPs since they are obtained under out-of-equilibrium conditions induced by the fast precipitation process. The differences from the expected values have been tentatively attributed to the plausible encapsulation of aqueous or hydroxyl iron complexes within the particles and the formation of secondary structures such as oligomers, as observed in previous reported NCPs [37,38,39]. In any case, and worth mentioning these results were fully reproducible over three different batches. Although different ratios of Pt:Fe were tested, the conditions indicated in the experimental procedure have been those that offered the best reproducibility. Based on the determined chemical formula, the Pt(IV)-complex payload value reached 76 wt% (≈30% in cisplatin content), more than 6-fold higher than most conventional metallodrug-loaded polymer carriers known to date (typically less than 10%) [40,41]. Finally, concerning dispersion of Pt-Fe NCPs in an aqueous solution, these nanoparticles showed remarkable colloidal stability in water and PBS simulating physiological environments. DLS and ζ-potential measurements indicated excellent monodispersion values and a slightly negative ζ-potential of −8.53 ± 0.418 mV (Figure S4), suggesting that the dispersion may remain stable for several days.

### 3.2. In Vitro Magnetic Resonance Imaging (MRI) Studies

The presence of high-spin Fe(III) ions within the Pt-Fe NCPs may also facilitate their use as MRI contrast agents, [42,43]. Then, concentration-dependent MR relaxometry experiments of the NCPs dispersed in 1% agarose PBS were performed. The addition of agarose ensured suitable dispersion of the nanoparticles and mimicked the viscosity and consistency of biological tissues [44]. From the resulting T1-weighted (T1w) and T2w imaging maps, relaxivity values of 0.4 and 21.6 mM^−1^ s^−1^ were obtained for the longitudinal (r1) and transversal (r2) parameters (Figure S5a), respectively (the resulting concentration-dependent T1w and T2w signal enhancement are shown in Figure S5b). Interestingly, the NCPs exhibited a low r1 value compared to the commercial gadolinium contrast agents (Gd-DTPA; 3.3 mM^−1^ s^−1^) [43] though comparable to values found in previously reported Gd-based NCPs, which is tentatively attributed to the limited water accessibility to the metal ions of the nanostructuration [45]. Though, Pt-Fe NCPs showed a noticeably high r2 value and, therefore, a remarkable r2/r1 ratio of 53.15, which indicates the potential of these nanoparticles to serve as a T2 contrast agent for MRI [46].

### 3.3. Drug Release Profile

**Figure 3:**
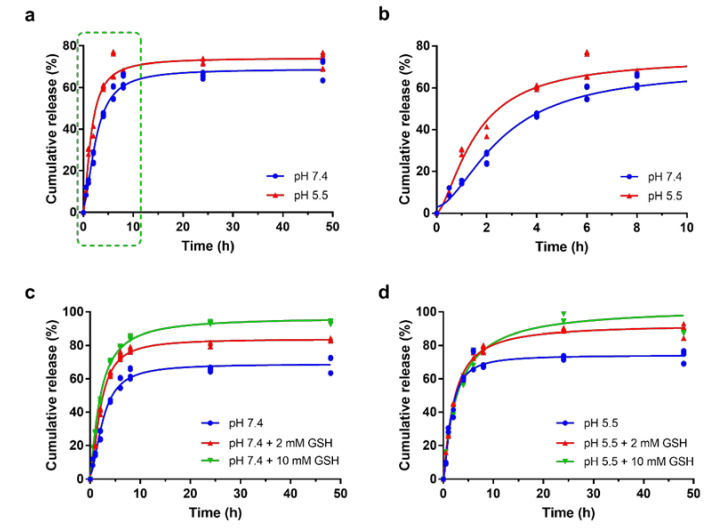
(**a**) Release profiles of Pt from Pt-Fe NCPs at 37 °C at pH 7.4 and 5.5 in PBS using dialysis method; (**b**) Inset of release showing the first 8 h of the whole release profiles (corresponding to green dashed line square shown in (**a**)). Each value represents the mean ± standard error (SE) of three independent experiments; Cumulative release of Pt from Pt-Fe NCPs in the absence or in the presence of GSH at (**c**) pH 7.4 and (**d**) pH 5.5. Each value represents the mean ± SE of three independent experiments.

Drug release profiles of the Pt-based species released from Pt-Fe NCPs were analyzed by ICP-MS in terms of concentration as a function of time (see Figure 3a,b). Pt-Fe NCPs were incubated in dialysis bags at 37 °C over 48 h in two different pH buffers (7.4 and 5.5), mimicking the physiological and mildly acidic intracellular conditions. Release curves at both pHs studied were typically bimodal, with a more pronounced release over the first 6 h and a sustained release over longer periods of time. Quantitatively, the Pt release at pH 5.5 emulating tumor microenvironments, turned out to be 60.45 ± 0.32% after 4 h and 72.43 ± 1.56% at 48 h of incubation. At physiological pH, the Pt release decreased to 46.78 ± 0.33% after 4 h and 67.92 ± 2.01% after 48 h of incubation, corroborating a notable pH influence in the release profile. This increased release at acidic pH was tentatively attributed to the lower stability of the coordinative bond between iron and catechol ligands, as previously described in related Fe-containing NCPs [27,47]. We have also evaluated the effect of glutathione (GSH) on the release profiles of Pt-Fe NCPs. Glutathiones is a natural compound that protects cells from oxidative damage and from the toxicity of xenobiotic electrophiles but may also enhance the intracellular reduction of Pt(IV) prodrugs into the corresponding Pt(II) active form. GSH concentrations in intracellular and extracellular environments have been found to range between 2–10 mM and 2–20 μM, respectively [48,49,50]. However, in our case, the more relevant value was that of GSH levels in the brain, reported to be 1–2 mM in normal conditions [51]. Therefore, the release experiments were repeated, now in the presence of GSH at two different concentrations of 2 mM and 10 mM. As shown in Figure 3, at pH 7.4 the Pt release was enhanced by GSH even at its lowest concentration of 2 mM, achieving values of 62.89 ± 0.54% and 83.54 ± 0.24% at 4 and 48 h, respectively. The aforementioned release was even higher in presence of 10 mM GSH. In this case, release values of 70.23 ± 0.28% and 93.49 ± 0.39% were obtained at 4 and 48 h, respectively. Similar trends were found at pH 5.5, demonstrating that Pt-Fe NCPs were both pH- and GSH-responsive.

### 3.4. Cytotoxicity Assays

The cytotoxicity of Pt-Fe NCPs was evaluated in vitro and compared to cisplatin and the precursor ligand complex **1** in murine GL261 GB cells and human cervical cancer cells HeLa. The well-established PrestoBlue method was used after 24 h and 72 h incubation (Figure S6). The half maximal inhibitory concentrations (IC_50_), referred to as the Pt concentration, were calculated using GraphPad Prism 7 (Table 1). After 24 h treatment, Pt-Fe NCPs showed similar cytotoxicity when compared to precursor complex **1** while toxicity proved considerably lower when compared to cisplatin. On the contrary, toxicity at 72 h was higher than the free ligand and comparable to cisplatin, tentatively attributed to the delay in nanoparticle degradation and, therefore, prodrug activation, as well as to different internalization and interaction mechanisms, as previously reported [26]. To confirm this possibility, further experiments were done.

### 3.5. Cell Internalization and DNA-Bound Pt

**Figure 4:**
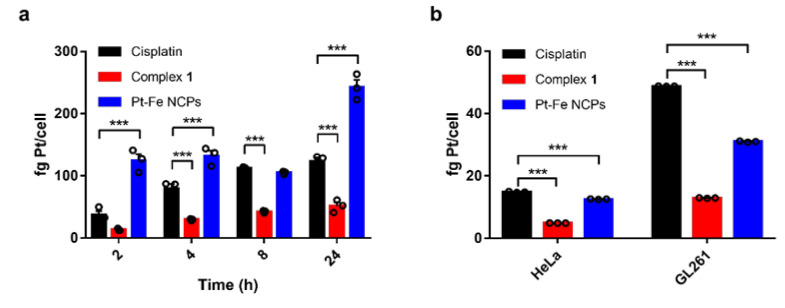
Cellular uptake determination of (**a**) total platinum content of GL261 cells after treatment with cisplatin, complex **1** and Pt-Fe NCPs at a concentration of 10 μM Pt along with time, and (**b**) DNA-bound Pt in HeLa and GL261 cells after exposure to cisplatin, complex **1**, and Pt-Fe NCPs for 24 h at a Pt concentration of 10 μM. Data represented as mean ± SE of three independent experiments. *** stands for *p* < 0.0001.

Cellular uptake of Pt-Fe NCPs, cisplatin, and the precursor ligand complex **1** was evaluated in GL261 GB cells at a Pt concentration of 10 μM and represented as fg of platinum per cell as measured by ICP-MS (Figure 4a). Results suggest that the cellular uptake of small molecules such as cisplatin and complex **1** increased in a time-dependent manner, while the uptake of Pt-Fe NCPs was significantly higher at every time point studied except 8 h, reaching a maximum internalization after 24 h. In addition to exhibiting different internalization mechanisms, the increased demand for iron as an essential nutrient for various biochemical activities associated with cell growth and proliferation may also contribute to the higher internalization of Pt-Fe NCPs [52]. Some researchers have reported that pathobiological events, most notably mTORC1 hyperactivity, which can be related to high-grade glioma, can increase Fe(III) uptake into cancer cells by regulating the activity of the transferrin receptor (TfR) in preclinical models [53].

However, and important to remark, the higher internalization of the NPs is not reflected in the toxicity but in a prolonged release. This imbalance urged us to figure out which was the fraction of the internalized Pt reaching the nucleus and hopefully crosslinking with the DNA to exert the cytotoxic effect. DNA-bound Pt was quantified using ICP-MS after 24 h internalization treatment for each formulation (Figure 4b). Interestingly, the amount of DNA-bound platinum in GL261 cells is higher than in HeLa cells, in agreement with the higher cytotoxicity observed after 24 h for all agents. Among them, the amount is much higher for cisplatin than for Pt-Fe NCPs. The nuclear pore complexes, the gatekeepers of the nucleus, only allow the diffusion of ions and species smaller than 10 nm [54,55,56], while molecules >40 kDa cannot pass through except by active transport [57]. Considering that the disassociation of the NCPs may take time to release the Pt(IV) prodrug and be further reduced to Pt(II) active form in order to bind to DNA, this fact can partially explain the reason why the increased cellular uptake did not result in augmented DNA-bound Pt or enhanced cytotoxicity in comparison to cisplatin.

### 3.6. ROS Production in GL261 cells

**Figure 5:**
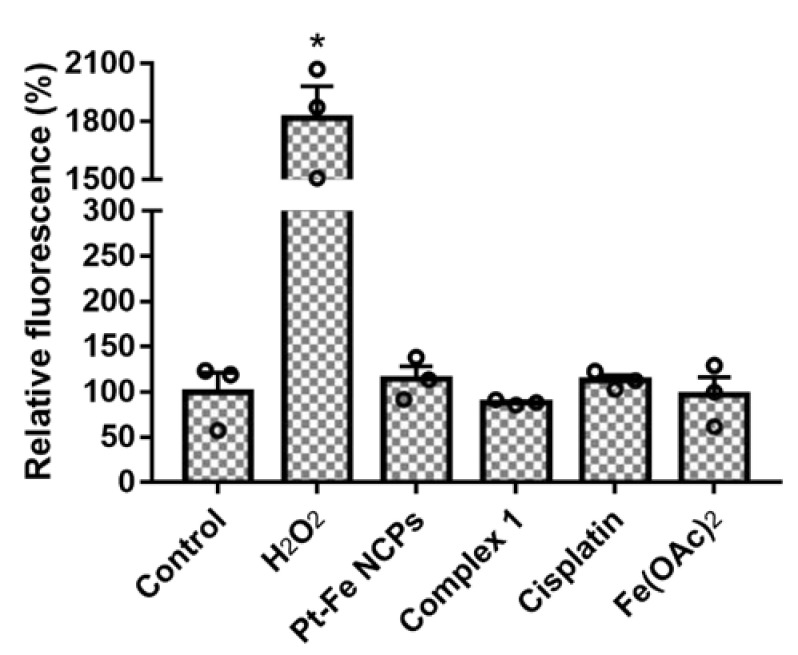
Determination of total ROS production in GL261 cells after 24 h of treatment with cisplatin, complex **1** and Pt-Fe NCPs at IC_50_ concentration concentrations, along with Fe(OAc)_2_ (100 µM), H_2_O_2_ (100 µM), or vehicle (control). Columns represent the mean fluorescence of oxidized DCFCDA. Data are expressed as means ± SE of three independent experiments. * denotes *p* < 0.05.

The excess of iron can induce oxidative stress through the generation of reactive oxygen species (ROS) in the central nervous system [58]. Therefore, we have investigated changes in total ROS production in GL261 GB cells using DCFCDA, a cell-permeable probe that is non-fluorescent when chemically reduced but becomes fluorescent after cellular oxidation and removal of acetate groups by cellular esterases. As shown in Figure 5, neither the platinum treatments nor Pt-Fe NCPs had a noticeable effect on ROS production in GL261 cells, while a 17-fold increase in total ROS production was detected after treatment with H_2_O_2_ (used as a positive control). These results suggest that the therapeutic effect of these NCPs could be rather attributed to the Pt(IV) contained in complex **1**, most likely reduced to the therapeutic active Pt(II) species upon internalization.

### 3.7. In Vivo Tolerability and Biodistribution via Intranasal Administration

**Figure 6:**
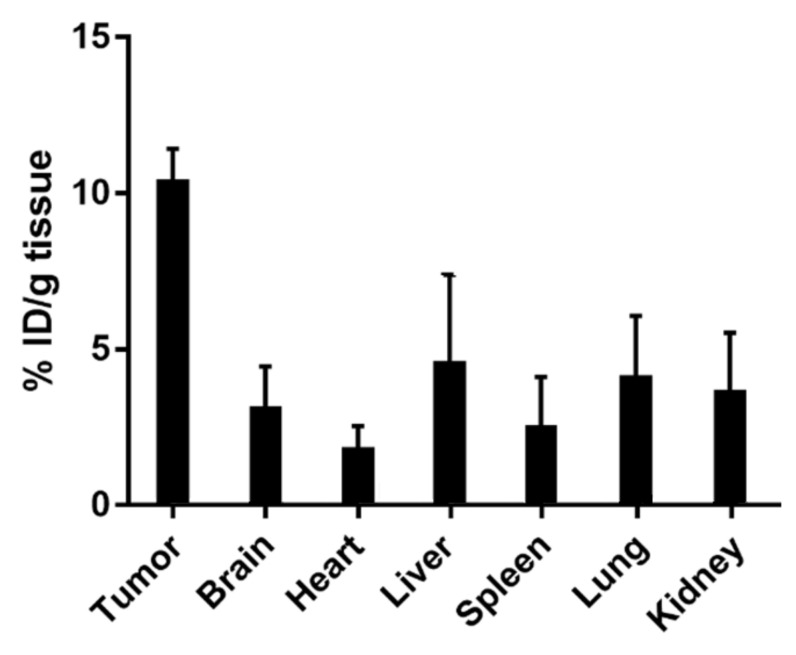
Biodistribution of Pt-Fe NCPs in mice organs 1 h after administration. Pt-Fe NCPs were given at a dosage of 1.5 mg/kg, *n* = 3. Data represented are mean ± SE from three independent experiments.

The safety and tolerability of Pt-Fe NCPs were assessed in a dose-escalation experiment [33] in wild type (wt) C57BL/6J mice with increasing doses (0.9, 1.2, and 1.5 mg Pt/kg body weight) administered for three consecutive weeks. Body weight, water/food consumption, and mouse behavior were assessed three times per week by veterinary staff for 4 weeks, and superimposed with normal body weight evolution of C57BL/6J, with no signs/symptoms indicative of suffering or toxicity detected (body weight evolution along time is shown in Figure S7). This was reinforced after the histopathological evaluation of tissues from administered mice used in tolerability studies, in which no significant morphological changes/abnormalities were found. The Pt-distribution in tumor and relevant organs such as liver, spleen, heart, lung, and kidney was determined by ICP-MS in GL261 GB-bearing mice 1 h after intranasal administration of the highest but still safe dosage (1.5 mg Pt/kg body weight). Very interestingly, and as shown in Figure 6, tumors retained most of the Pt (10.34% initial dose (ID) per gram of tissue) with a lower accumulation in the non-affected brain (differential accumulation), which was in turn comparable to other examined organs (see Supporting Information Section S5, Table S2). These successful results encouraged us to finally assess the In vivo anticancer efficacy. It is worth remembering that an exhaustive study of Pt-Fe NCPs biodistribution was beyond the scope of this work and may be the subject of further studies whenever suitable doses and effect mechanisms are fully elucidated.

Even in the presence of the aforementioned differential accumulation, unfortunately, we were not able to detect enhancement in in vivo T1w and T2w MRI signals in spite of several attempts (data not shown). This might be most likely due to the insufficient iron concentration reaching the brain after intranasal administration of the maximum tolerated dose (1.5 mg of Pt and 0.93 mg Fe per kg of body weight). This would explain why previous studies on iron-based NCPs administered intravenously at higher doses (ca. 0.4 mmol of Fe per kg of body weight ≈23 mg/kg) did show contrast [42].

### 3.8. In Vivo Anticancer Efficacy

The in vivo efficacy of Pt-Fe NCPs was evaluated by measuring the time-dependent tumor evolution using T2w MRI in orthotopic GL261 GB-bearing mice (*n* = 8) and using an every 6-day interval following an immune friendly schedule (Figure S8). This protocol was named immune-enhancing metronomic schedule and proved to achieve excellent results with TMZ and also immunotherapy [59,60]. Untreated mice (*n* = 7) were used as controls. The IN administration dose was 1.5 mg Pt/kg body weight, which was the highest safe dose in the tolerability assessment and proved to be well-tolerated for wt mice. In any case, mouse body weight and welfare status were recorded twice a week in order to assess disease evolution and check for any suffering signs.

**Figure 7:**
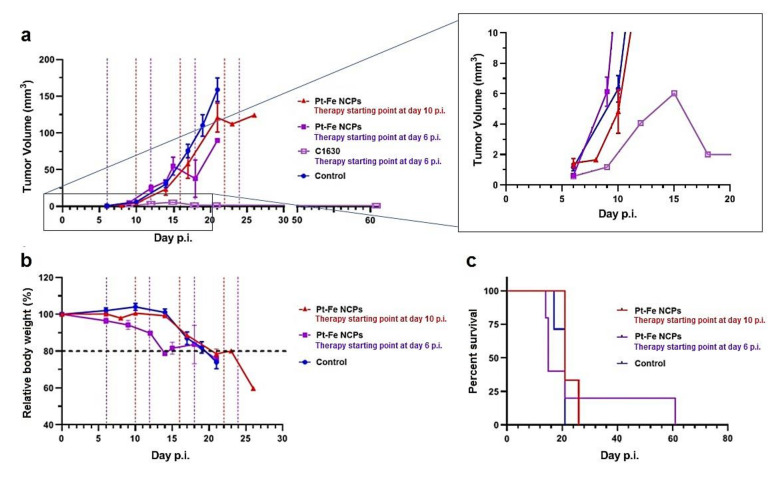
(**a**) Tumor volume evolution measured through T2w MRI. Note that a zoomed image of the case C1630 (cured case) in the period 0–20 days p.i. is shown apart on the right to ensure better visualization of the group evolution, (**b**) Mice relative body weight and (**c**) Kaplan–Meier survival curves for control mice (*n* = 7, blue line), mice administered with Pt-Fe NCPs starting at day 10 p.i. (*n* = 3, red line) and starting at day 6 p.i. (*n* = 5, purple line). Note that graph in (**a**) shows mean values and it is restricted to 30 days for comparison purposes, without extending the graph to encompass the cured case. See Supplementary Figures S9 and S10 for checking evolution of C1630, the cured case in this cohort.

The first set of experiments was launched on day 10 post-implantation (p.i.) with an initial average tumor volume of 6.32 ± 2.31 mm^3^ and no significant differences from volumes measured in control mice, 4.83 ± 1.43 mm^3^. In this case, only one of the treated tumors exhibited a slower evolution. The average calculated doubling time (DBT), defined as the time needed for a given tumor to duplicate its volume [61], merely increased an 18% (2.2 ± 0.3 vs. 2.6 ± 0.4 days for control and treated mice, respectively), reflecting only a modest arrest in tumor growth not enough to halt tumor progression. In fact, there was no significant improvement in overall survival (19.9 ± 2.0 days and 22.7 ± 2.9 days for control and treated with Pt-Fe NCPs, respectively. The overall evolution of mouse body weight during treatment is shown in Figure 7b, and the resulting Kaplan–Meier curves can be seen in Figure 7c. Then, we took a step back and considered previous results describing how the initial p.i. day, i.e., initial tumor volume, was determinant in achieving a suitable response to therapy. [62,63].

The next treatment round was then planned from an earlier starting point (day 6 p.i.), with a mice cohort bearing smaller tumors than in the first set of experiments, below 1 mm^3^ (0.63 ± 0.9 mm^3^). In this way, we did manage to prolong the average survival time in comparison with control tumors (Figure 7), although with an excessive dispersion (25.2 ± 20.2 days). This is in line with the well-known heterogeneity of GB, even when dealing with these types of preclinical induced tumors generated under controlled conditions. However, more relevant was the case of cured mice C1630 (Figure 7a), showing the disappearance of a tumor mass with only a remnant tissue abnormal region detected by T2w MRI at day 18–21 p.i. (Figure S9). Moreover, mouse C1630 did not exhibit any health status alteration until day 61 when it was found dead, and postmortem MRI did not suggest any tumor regrowth, pointing that brain tumor relapse was not the cause explaining animal death.

Overall, 1 out of 5 (i.e., 20%) of the studied cohort was cured with this protocol, and the tumor growth arrest became more evident after the second therapeutic cycle at day 12 p.i. (DBT increased to an average of 3.3 ± 1.4 days, presenting a trend to significance in comparison with control mice (*p* = 0.058)). It is worth noting that 60% of the individuals (*n* = 3) presented shortened survival in comparison to controls. This is probably a result of a mixed effect due to early starting, which may also trigger unexpected early toxicity due to off-target effects. This effect is worth future investigation since it seems to be more evident in tumor-bearing mice. The investigated wt mice did not present harmful effects after increasing administered doses. Figure S10 illustrates the two different growth rate evolution observed in extreme cases, C1630 (cured case) and C1647 (non-responding case). Unfortunately, even with a cohort size that proved representative of GB heterogeneity according to our previous experience, we were not able to reach statistical significance between control and treated mice. This points out that there are still missing pieces to be unraveled in this experimental problem, and we cannot discard that a cohort enlargement would be needed to reach significance, and will be the object of future dedicated work.

## 4. Discussion

### 4.1. Advantages of Pt(IV) Prodrug Nanoformulation

In vitro assays determined that these Pt(IV) nanoparticles exhibited a notable controlled-release cytotoxic effect against cancer cells, with slightly higher IC_50_ values at 24 h but fully comparable to the gold standard Pt(II) drug cisplatin at 72 h. This lag does not come from differences in cell internalization, since Pt-Fe NCPs exhibit improved intracellular uptakes than cisplatin, but it could be most likely related to the slower release and activation period needed for the Pt(IV) prodrug activation. DNA-bound Pt confirmed this clue since platinum contents in GL261 GB cells after 24 h of exposure were much higher than that of Pt-Fe NCPs. Finally, this remarkable drug release effectiveness is associated with the minimization of drug-induced side effects. As reported in previous studies, Pt(IV) prodrugs have been mainly used to counteract cisplatin resistance and nephrotoxicity effects. While in their Pt(IV) oxidation state, side effects are considerably minimized, and they are reduced to the antitumoral-active Pt(II) form inside the cells [64]. Moreover, encapsulation of Pt(IV)-prodrugs has been shown not only to facilitate the BBB crossing but also to increase cellular uptake and cytotoxicity in GB cells without compromising toxicity [65,66].

### 4.2. In Vivo Efficacy Is Not Directly Related to the Paradigm “More Is Better”: Words of Caution

An immune-respectful therapeutic schedule has been shown to improve outputs with chemotherapeutic approaches [59,67], immunotherapy alone or in combination with chemotherapy [60], and targeted therapy (e.g., CK2 inhibition) [68], even inducing “immune memory” in a certain percentage of cases [59]. In the case of GB, it is usually considered an immunologically “cold” tumor, with limited clinical response to the therapy [69], but it can be converted to an immunologically “hot” tumor when immunogenic cell death is triggered [70]. Therefore, therapeutic approaches able to produce immunogenic cell death/damage, eliciting host immune response could increase therapy efficacy and lead to immune memory [71]. In other words, triggering immunogenic cell death while respecting the host immune system during therapy has been widely acknowledged as a relevant aspect to ensure proper outcome [72,73,74], and platinum compounds are not an exception [65,75,76]. Platinum derivatives were described to induce immunogenic cell death [77], namely through calreticulin exposure [78] or ATP release [79]. For instance, a recent report demonstrated that CED cisplatin induced cure in immunocompetent GL261 models, but failed to cure immunocompromised NSG mice with intracranial tumors, revealing that the therapeutic effects of CED cisplatin were immune-dependent [80]. This provides an important hint on how the administration schedule of chemotherapeutic agents should be in harmony with the immune cycle to recruit the host immune system into the battle against GB.

In the present work, an optimized therapy in immune-enhancing metronomic schedule (IMS) starting at day 6 post-implantation resulted in significant inhibition of tumor growth in GL261 orthotopic models. Namely, we found that 1 out of 5 (20%) animals were cured, and the same rate presented partial response with transient growth arrest, resulting in enlarged average survival time. Authors are aware that this is a small cohort, and further studies are needed to corroborate this trend, but also consider that these are promising preliminary results. It is worth noting that the fact that one out of five (20%) of the cohort administered from day 6 p.i. got cured is not a subjective perception. The protocol for considering “cured” mice was previously described [81], and it depends on reliable initial detection of a tumor mass and coherent growth before therapeutic administration. The GL261 tumor is a very aggressive tumor model which, without treatment, will kill implanted animals in a range of ca 19–23 days after implantation. In this sense, tumor regression and disappearance are considered a cure. Moreover, the cured animal was followed for signs/symptoms of tumor relapse, and once it was found dead, postmortem MRI did not point to relapsing tumor growth, which discards GB as the cause for the death of the cured animal. Furthermore, the authors considered that the disappointing results obtained from the cohort with treatment starting at day 10 p.i. discouraged increasing this specific group, saving efforts for groups following more promising therapeutic schedules.

Nevertheless, the authors recognize that the role of host immunity in our in vivo studies should still be elucidated by further experiments in the future to investigate the possible modulation of immunogenic effects. The authors are also aware that suitable clinical translation would require the use of human-derived tumor-bearing mice. However, the regular patient-derived xenografts do not seem to be the best option in this scenario, since these models are developed over immunosuppressed mice and do not recapitulate intact host immune system, hence lacking part of the response derived from the immunogenic potential of agents such as platinum-derived ones. In future studies, the use of humanized xenografts may be contemplated as a suitable option to further corroborate satisfactory results in a human-related environment.

A word of caution should be raised regarding toxic effects from platinum derivatives that might not be neglected, such as neurotoxicity [82], nephrotoxicity, cardiotoxicity, or hepatotoxicity [83]. This suggests that a balanced combination of dose and schedule of administration should be achieved in order to get optimum outcomes, both in preclinical and clinical settings. Clarification of the reasons behind differences in possible toxic effects in tumor-bearing mice and wt mice is still required since Pt-Fe NCPs seemed to lack harmful effects in the latter. In this study, the cured case treated with Pt-Fe NCPs did not present signs of tumor relapsing, which was encouraging information, although no information about immune memory was gathered in this case. This toxic effect is not new and was already reported in a previous study evaluating the in vivo anticancer efficacy of cisplatin-loaded polybutylcyanoacrylate (PBCA) NPs in C6 GB bearing-rats via intraperitoneal injection [44]. The treatment started as early as 2 days p.i. at a dose of 1 mg/kg (referred to cisplatin), being administered every 3 days up to a total of 4 doses. However, the mean survival time of mice treated with cisplatin-PBCA NPs was 17.5 days compared to 19.6 days for the cisplatin-free group, indicating the possible prevailing toxicity over beneficial effects induced by the nanoformulation, which was confirmed by histopathological studies showing liver damage. In this case, the nanosized formulation of cisplatin did not show effects in average survival or tumor volumes and intense-dosing schedule via intraperitoneal route even with a potentially small starting volume, which would agree with findings reported in this work.

## 5. Conclusions

The results gathered in this work open a future path for investigation of IN platinum nanoderivatives for brain tumor treatment, overcoming BBB permeability challenges, even in high-grade brain tumors such as GB. Indeed, this study has revealed that the nanostructuration of Pt(IV) prodrugs could reduce the possibility of cytotoxic side effects due to the precisely controlled release and the time necessary for the activation of the Pt(IV) prodrug. Thus, these novel Pt(IV)-based nanoparticles exhibited a notable controlled-release cytotoxic effect fully comparable to cisplatin at relatively long times (72 h), mostly related to the slower release profile and activation period needed for the Pt(IV) prodrug activation to the antitumoral-active Pt(II) from inside the cells. This would represent a clear advantage with respect to the use of cisplatin since this remarkable drug release effectiveness is associated with the minimization of drug-induced side effects such as the reduction in cisplatin resistance or nephrotoxicity effects. Moreover, the resulted nanoformulation was suitable for intranasal administration to mice bearing GB tumors, and the anticancer activity in vivo was confirmed through noninvasive follow-up with T2w MRI and tumor volume measurements derived from imaging. Our overall results with mice cohorts administered with Pt-Fe NCPs, using an optimized immune-enhancing metronomic schedule therapy in GL261 orthotopic models, suggest that a dose reduction or more control in the release profile may be needed to ensure that toxic effects do not surpass the beneficial effects, especially in the frame of an early time point for treatment starting. The complex immunosuppressive GB environment will probably request a multi-approach treatment, in which a combination panel would be the best proposal to achieve suitable outcomes, in which the IN Pt-Fe NCPs would be a relevant player.

## Figures and Tables

**Table 1:** Comparative study of IC_50_ values for Pt(IV)-NCPs, Complex **1**, and cisplatin after 24 and 72 h of incubation.

IC_50_ (µM) ^a^				
	Cell Line (24 h)		Cell Line (72 h)	
Compound	HeLa	GL261	HeLa	GL261
Pt-Fe NCPs	31.45 ± 1.10	13.48 ± 0.90	2.56 ± 0.63	2.00 ± 0.18
Complex **1**	29.94 ± 1.04	17.40 ± 1.08	1.85 ± 0.36	4.17 ± 0.12
Cisplatin	15.98 ± 1.04	5.61 ± 0.28	2.34 ± 0.30	2.16 ± 0.26

^a^ The IC_50_s of the compounds against different cell lines were determined by PrestoBlue assay. Each value represented the mean ± SE of 3 independent experiments.

## Data Availability

Data are available on the request from the corresponding author.

## Supplementary Materials

The following are available online at https://www.mdpi.com/article/10.3390/nano12071221/s1, Figure S1: Powder XRD (a) and EDX (b) of Pt-Fe NCPs, Figure S2: Mössbauer spectra of Pt-Fe NCPs, Figure S3: FT-IR spectra of vomplex 1 and Pt-Fe NCPs, Figure S4: DLS and ζ-potential of Pt-Fe NCPs, Figure S5: In vitro T1/T2 relaxometry studies for Pt-Fe NCPs, Figure S6: Cytotoxicity of Cisplatin, complex **1** and Pt-Fe NCPs against HeLa and GL261 cells, Figure S7: Tolerability assessment of mice for Pt-Fe NCPs, Figure S8: Experimental treatment schedule and representative T2w tumor images, Figure S9: T2w high-resolution MRI images, Figure S10: Tumor volume evolution of two representative cases, Table S1: Elemental analysis and ICP-MS data of Pt-FeNCPs, Table S2: Biodistribution study of Pt-Fe NCPs in mice.
