# Supplementary material for: Intranasal Administration of Catechol-Based Pt(IV) Coordination Polymer Nanoparticles for Glioblastoma Therapy
Source: Nanomaterials (Basel). 2022 Apr 5;12(7):1221. doi: 10.3390/nano12071221 (PMC9003391; doi:10.3390/nano12071221)
Supplement: Supplementary file 1 [file nanomaterials-12-01221-s001.zip › nanomaterials-1634351-supplementary.pdf]

nanomaterials

Supporting Information

Intranasal Administration of Catechol-Based Pt(IV) Coordination Polymer Nanoparticles for Glioblastoma Therapy

Xiaoman Mao [1], Pilar Calero-Pérez [2,3], David Montpeyó [4], Jordi Bruna [5], Victor J. Yuste [3,6], Ana Paula Candiota [2,3,4,*], Julia Lorenzo [3,4,*], Fernando Novio [1,7,*] and Daniel Ruiz-Molina [1]

[1] Catalan Institute of Nanoscience and Nanotechnology (ICN2), CSIC and BIST, Campus UAB, Cerdanyola del Vallès, 08193 Barcelona, Spain; mmchong7@zju.edu.cn (X.M.); dani.ruiz@icn2.cat (D.R.-M.)
[2] Centro de Investigación Biomédica en Red: Bioingeniería, Biomateriales y Nanomedicina, Cerdanyola del Vallès, 08193 Barcelona, Spain; pilar.calero@uab.cat
[3] Departament de Bioquímica i Biologia Molecular, Universitat Autònoma de Barcelona, Cerdanyola del Vallès, 08193 Barcelona, Spain; victor.yuste@uab.cat
[4] Institut de Biotecnologia i de Biomedicina, Universitat Autònoma de Barcelona, Cerdanyola del Vallès, 08193 Barcelona, Spain; david.montpeyo@uab.cat
[5] Neuro-Oncology Unit, Bellvitge University Hospital-ICO (IDIBELL), Avinguda de la Gran Via de l'Hospitalet, 199-203, L'Hospitalet de Llobregat, 08908 Barcelona, Spain; jbruna@bellvitgehospital.cat
[6] Institut de Neurociències, Facultat de Medicina, Universitat Autònoma de Barcelona (UAB), Campus UAB, Cerdanyola del Vallès, 08193 Barcelona, Spain
[7] Departament de Química, Universitat Autònoma de Barcelona (UAB), Campus UAB, Cerdanyola del Vallès, 08193 Barcelona, Spain
* Correspondence: anapaula.candiota@uab.cat (A.P.C.); julia.lorenzo@uab.es (J.L.); fernando.novio@icn2.cat (F.N.); Tel.: +34-935814126 (A.P.C.); +34-935868957 (J.L.); +34-937373630 (F.N.)

S1. Characterization of Pt-Fe NCPs

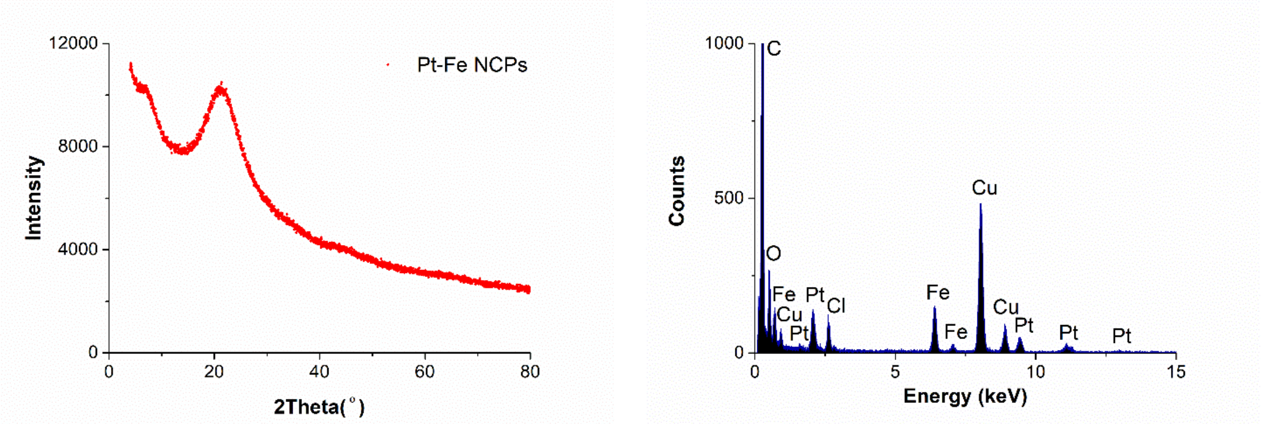

(a) (b)

Figure S1. Powder XRD (**a**) and EDX (**b**) of Pt-Fe NCPs.

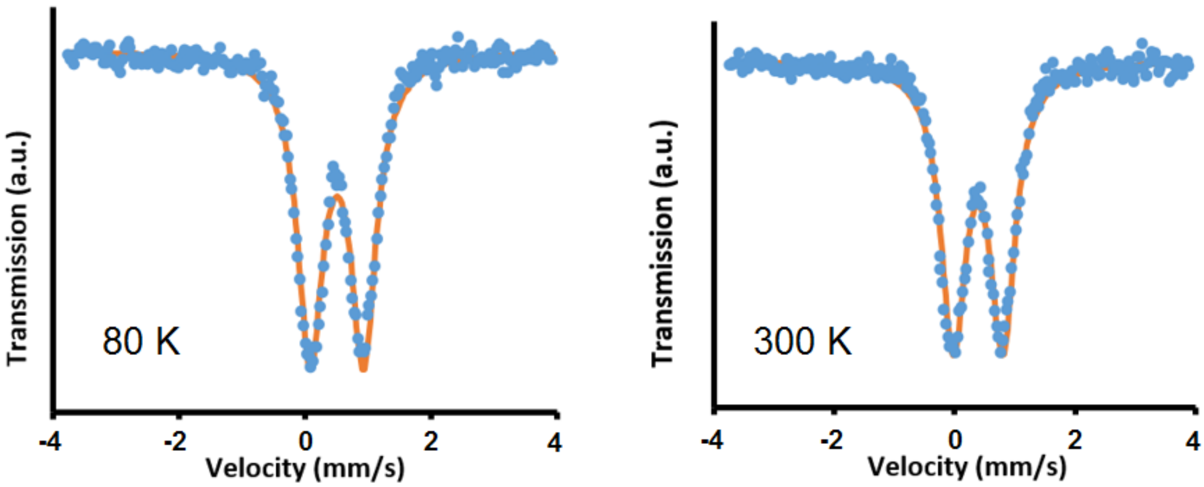

Figure S2. Representative Mössbauer spectra of Pt-Fe NCPs at 300 K and 80 K. Experimental data (blue dots), and computation fitted (orange line) spectra. The hyperfine parameters of the fitting of Mössbauer at 300 K showed the isomer shift relative to the metallic iron (δFe), quadrupolar splitting (ΔEq) and the full width at half maximum (Γ). The spectrum was fitted to a single doublet with a ΔEq = 0.81 ± 0.04 mm/s and Γ = 0.32 mm/s. The fitting was centered at an isomeric shift δ = 0.39 ± 0.03 mm/s.

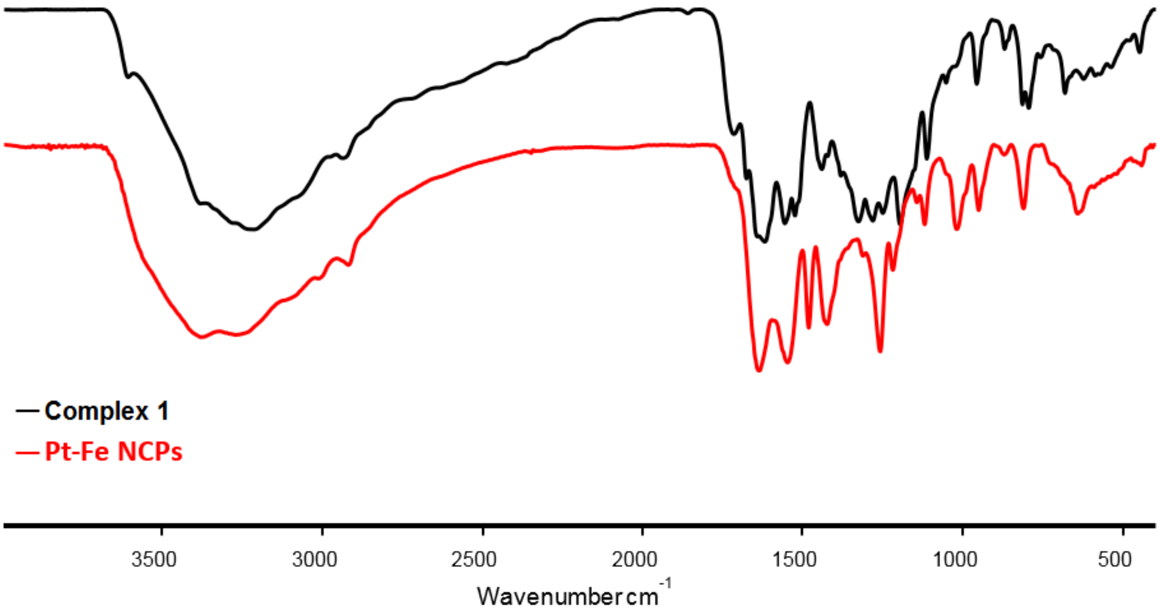

Figure S3. FT-IR spectra of the Pt(IV) prodrug (Complex **1**) and Pt-Fe NCPs.

Table S1. Experimental composition (Exp. % wt) obtained by elemental analysis and ICP-MS, and correlation with the theoretical values (Cal. % wt) for the proposed chemical formula [(Complex **1**)$_1$Fe$_3$(OH$^-$)$_5$]$_n$.

	C	H	N	Pt	Fe
Exp. wt.%	28.63	2.54	5.79	18.87	15.13
Cal. wt.%	27.27	3.72	5.30	18.45	15.83
Error %	0.99	1.18	0.69	0.42	0.7

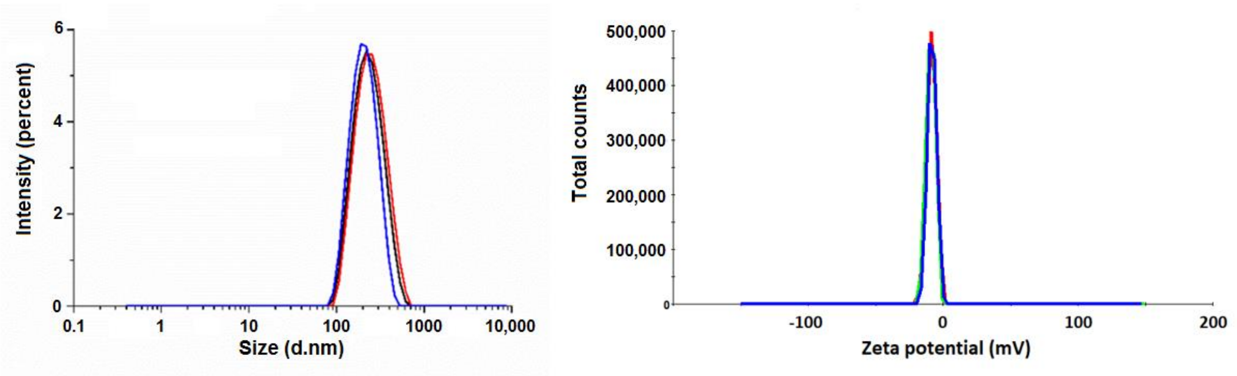

Figure S4. Representative size and ζ-potential of Pt-Fe NCPs by DLS obtained in PBS for three days.

S2. In Vitro Magnetic Resonance Imaging (MRI) Studies

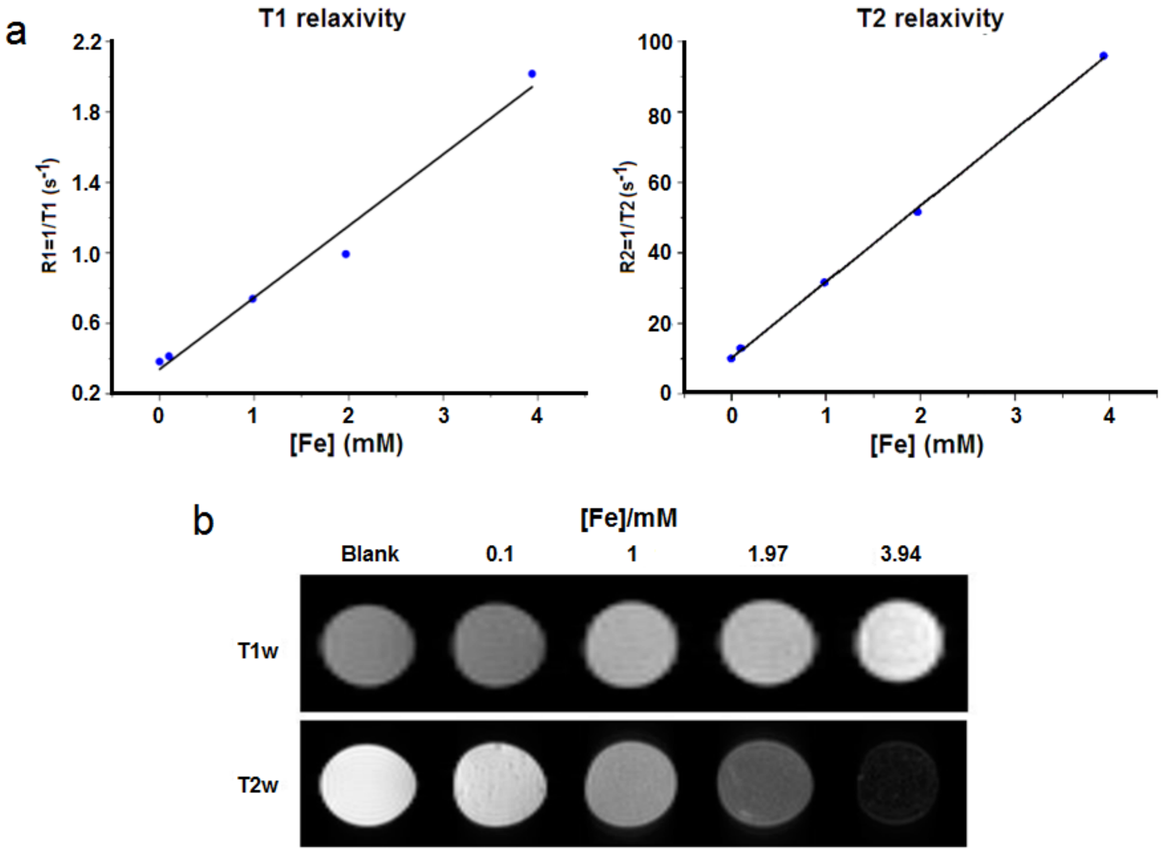

Figure S5. (**a**) In vitro T1/T2 relaxometry studies for Pt-Fe NCPs at different Iron concentrations. (**b**) T1w and T2w MRI obtained with Pt-Fe NCPs which were suspended in PBS containing 1% agarose at pH 7.4 at different concentrations (0, 0.1, 1, 1.97 and 3.94 mM, referred to Fe concentration).

S3. In Vitro Anticancer Studies

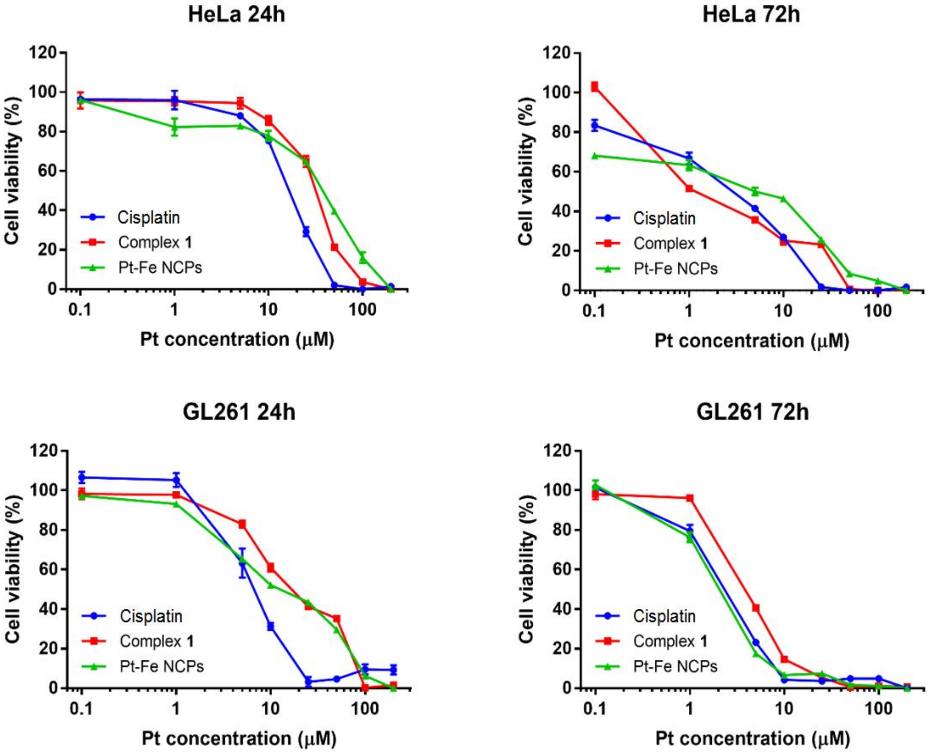

Figure S6. Cytotoxicity of Cisplatin, complex **1** and Pt-Fe NCPs against HeLa and GL261 cells at 24 h and 72 h. All data represented mean ± SE of three independent experiments.

S4. In Vivo Tolerability Assays

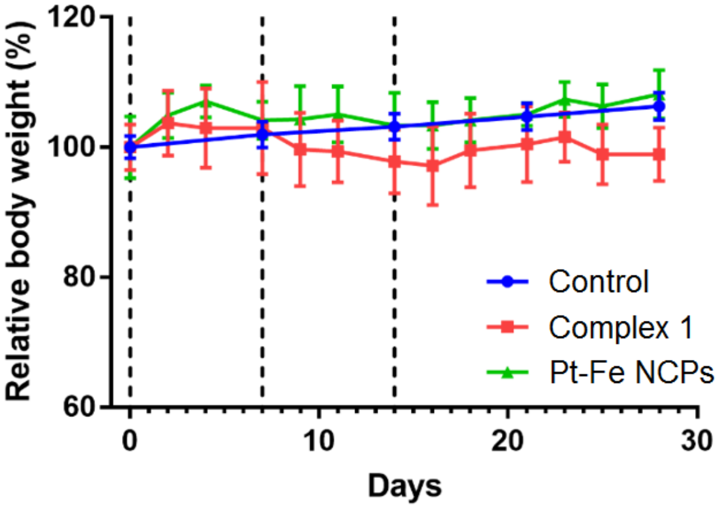

Figure S7. Tolerability assessment in female wt C57BL/6 mice for Pt-Fe NCPs over 4 weeks. Mice were administered with increasing doses from 0.9, 1.2 to 1.5 mg Pt/kg body weight via I.N. once a week, n = 3. For control group, n = 40, data adapted from Jackson Laboratory (https://www.jax.org/jax-mice-and-services/strain-data-sheet-pages/body-weight-chart-000664 (accessed on 24 March 2022)). Data represented as mean ± SE. Vertical dash lines indicated days for administration.

S5. Experimental In Vivo Treatment Schedule

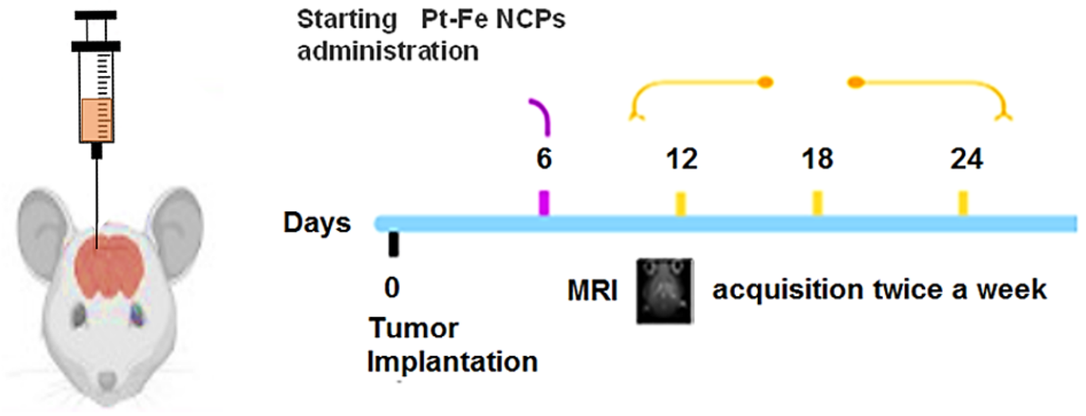

Figure S8. Experimental treatment schedule (starting point in purple and further administration points in yellow) and representative T2w tumor images of cases on the therapy starting day.

S6. In Vivo Biodistribution Study

Table S2. Biodistribution study of Pt-Fe NCPs in mice. Dose = 1.5 mg Pt/kg, biodistribution time = 1 h, $n = 3$.

	**Pt Retention in Mice (% dose/g tissue)**
Tumor	10.34 ± 1.09
Brain	3.06 ± 1.40
Heart	1.75 ± 0.78
Liver	4.50 ± 2.89
Spleen	2.44 ± 1.68
Lung	4.06 ± 2.0
Kidney	3.58 ± 2.0

S7. MRI Monitoring of Tumor Evolution During Treatment: Illustration of Two Representative Cases

C1630: cured case. The evolution was fast at initial stages, then the tumor growth got arrested after day 15 p.i. After reaching a clear volume of 6.03 mm^3 on day 15 p.i., tumor mass decreased and disappeared. This case accomplished criteria for cured cases as described in PMID: 31926117 [1], when abnormal mass detection by MRI became no longer possible, or the volume remained stable (usually less than 2 mm^3). In this case and following the agreed protocol, treatment was halted and mouse inspected for abnormal behavior or body weight losses that could be indicative of tumor relapsing in addition to MRI monitoring twice a week. This mouse was found dead on day 61 p.i., and postmortem MRI did not suggest any relapsing tumor mass. See figure S9 and figure S10 below.

C1647: unresponsive case. As opposed to the previous, this case presented fast evolution without signs of response, not even transient response. Volumes and survival were comparable to control (untreated) cases.

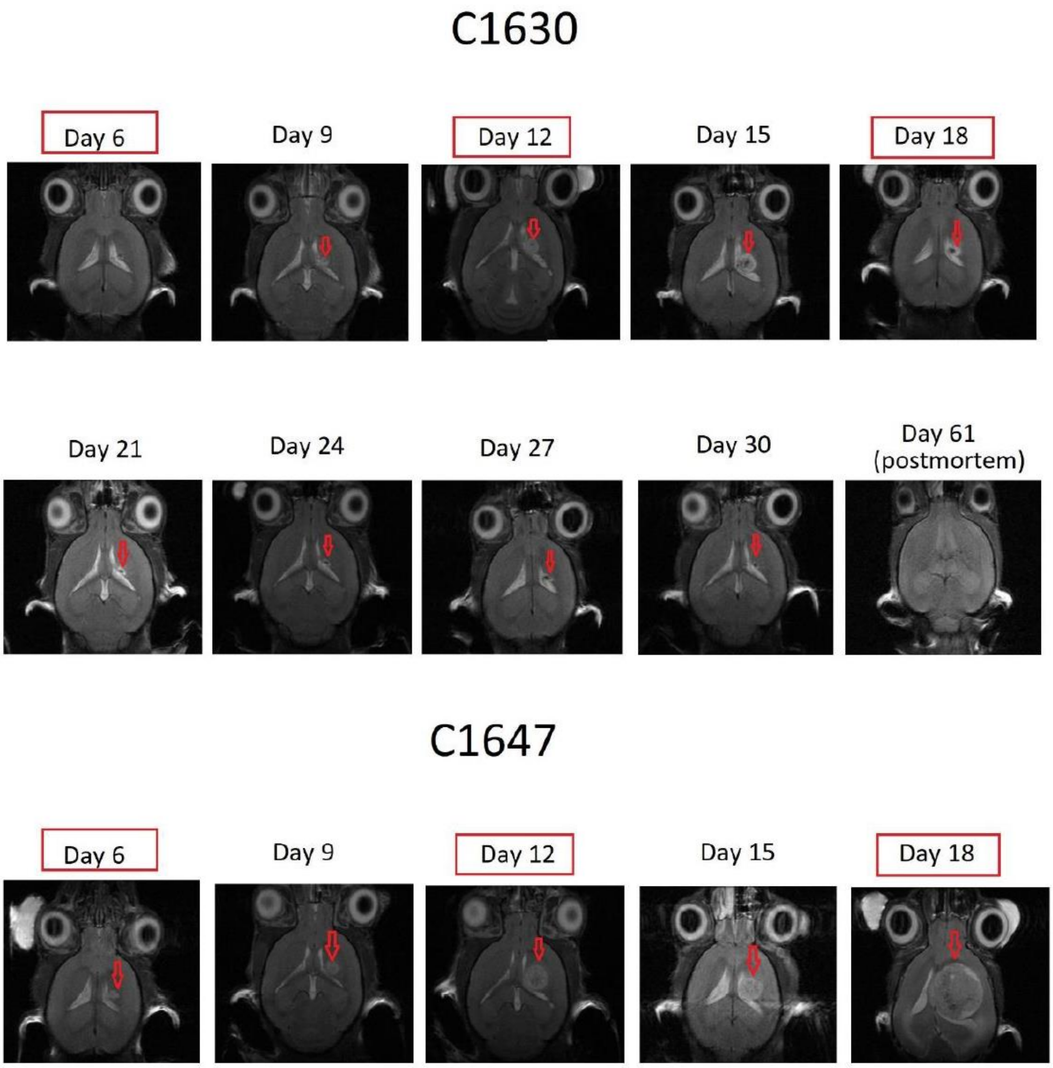

Figure S9. T2w high-resolution MRI acquired at 7 T for animals C1630 and C1647 (treated with IMS protocol starting on day 6 p.i.) at the different time points assessed. The tumor was highlighted with a red arrow. From day 21–24 p.i. mouse C1630 was declared cured and tumor reduced to a visible but stable scar with no evolution in further follow-up. This animal was found dead at day 61 p.i. and a post-mortem fast MRI exploration was performed in order to discard presence of visible tumor. Mouse C1647 did not respond to treatment and presented fast growth evolution. Red squares indicate therapeutic administration points.

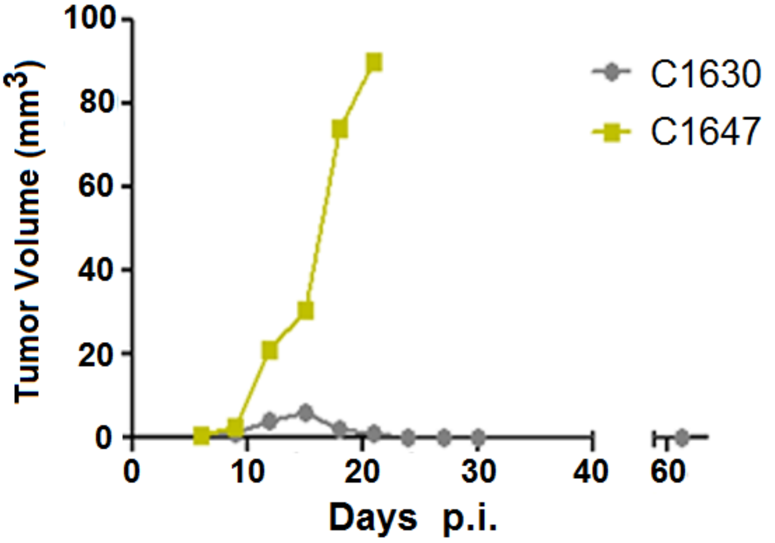

Figure S10. Tumor volume evolution of two representative cases in the group treated with Pt-Fe NCPs at day 6 p.i.

References:

1. Wu, S.; Calero-Pérez, P.; Villamañan, L.; Arias-Ramos, N.; Pumarola, M.; Ortega-Martorell, S.; Julià-Sapé, M.; Arús, C.; Candiota, A.P. Anti-Tumour Immune Response in Gl261 Glioblastoma Generated by Temozolomide Immune-Enhancing Metronomic Schedule Monitored with MRSI-Based Nosological Images. *NMR Biomed.* **2020**, *33*, e4229.
